# Interleukin-2 and Tretinoin for Myeloproliferative Neoplasms and to Target Type 1 Calreticulin-Driven Neoplasms: Advancements in Immune Regenerative Medicine

**DOI:** 10.3390/ijms27062814

**Published:** 2026-03-20

**Authors:** Dipnarine Maharaj, Wen Zhang, Kawaljit Kaur, Jacqueline Gouvea

**Affiliations:** 1 The Maharaj Institute of Immune Regenerative Medicine, Boynton Beach, FL 33437, USA; zhang.wen81@gmail.com (W.Z.); jgouvea@bmscti.org (J.G.); 2 ImmuneLink, LLC, Riverside, CA 92508, USA; drkawalmann@g.ucla.edu

**Keywords:** stem cells (SCs), cancer stem cells (CSCs), calreticulin (*CALR*) mutation, personalized, precision, immunotherapy, targeted therapy, tretinoin, cytokines, interleukin-2 (IL-2), natural killer (NK) cells

## Abstract

Stem cells, also known as progenitor cells, can differentiate into specialized cells for specific tissues. Genetic mutations and epigenetic changes may cause normal stem cells to become cancer-initiating cells. Research indicates that cells acquiring a mutation for myeloproliferative neoplasm (MPN) are likely to be long-term hematopoietic stem cells (LT-HSCs) at the top of the hematopoietic hierarchy. Natural killer (NK) cells play a crucial role in combating cancer by targeting and eliminating cancer stem cells (CSCs) while promoting their maturation. NK cells do this through direct lysis of CSCs or by releasing cytokines like interferon-gamma (IFN-γ) and tumor necrosis factor-alpha (TNF-α), which inhibit tumor growth and metastasis by driving differentiation of CSCs. Interleukin-2 (IL-2) enhances the activity of CD4+ and CD8+ T cells and boosts NK cell cytotoxicity. This study highlights a case of MPN with a more clinically aggressive Type 1 calreticulin (*CALR*) mutation, where a combination of low-dose IL-2 immunotherapy and targeted therapy with oral tretinoin (all-trans retinoic acid, ATRA, a vitamin A derivative) improved immune cells, particularly NK-cell-mediated destruction of malignant cells, reduced *CALR* mutation levels to undetectable, and alleviated disease symptoms. The aim is to offer a new, low-toxicity personalized treatment strategy that eradicates cancer-initiating stem cells, reduces side effects, and provides an option for patients with limited conventional therapy alternatives.

## 1. Introduction

Stem cells (SCs) have become a key focus in recent research; they have a remarkable ability to self-renew and differentiate into mature cells specific to various tissue types [1]. Hematopoietic stem cells (HSCs), as multipotent precursors, can self-renew and regenerate the diverse cells of the blood-forming system [2]. The initial cells that acquire a myeloproliferative neoplasm (MPN)-initiating mutation are long-term hematopoietic stem cells (LT-HSCs), located at the top of the hematopoietic hierarchy [3,4,5]. Unlike acute leukemia, where myeloid progenitor cells gain abnormal self-renewal, MPN initiation and progression depend on cells within the immunophenotypically defined LT-HSC compartment [6,7,8,9]. The myeloproliferative leukemia virus oncogene (MPL), also known as the thrombopoietin receptor gene, plays a crucial role as a growth factor receptor in MPN development [10]. When activated, MPL signaling triggers molecular events that give MPN stem cells a clonal advantage in the bone marrow [11]. MPNs share clinical and biological traits, including activation of the Janus Kinase–Signal Transducer and Activator of Transcription (JAK-STAT) pathway, which regulates vital cellular functions like growth, survival, and differentiation, particularly in blood-forming HSCs [12]. However, MPNs exhibit diverse clinical phenotypes influenced by mutations in the disease-initiating HSCs and their clonal progeny, contributing to disease variability [12].

The MPN phenotype is shaped in part by acquired driver mutations, with calreticulin (*CALR*) and MPL mutations typically associated with essential thrombocytopenia (ET) or myelofibrosis (MF) phenotypes, but not Polycythemia Vera (PV) phenotypes [13]. These driver mutations in MPN stem cells are believed to play a critical role in initiating, maintaining, progressing, and resisting drugs in neoplasms [13]. *CALR* mutations, such as Type 1 (52-bp deletion) or Type 2 (5-bp insertion), produce a mutant calreticulin protein that improperly activates the thrombopoietin receptor MPL, leading to persistent *JAK-STAT* signaling and excessive megakaryocyte proliferation [14]. Patients with Type 1 CALR mutations experience a more aggressive disease course with rapid progression to acute myeloid leukemia (AML) compared to individuals with Type 2 mutations [15]. CALR mutations are found in NK cells, along with other blood cells, altering their biology. Some studies show NK cell dysfunction in MPN patients, potentially hindering anti-leukemic responses. Type 1 *CALR* mutations deregulate more pathways in lymphoid cells, including NK cells, than Type 2 mutations [16].

Treating MPN stem cells requires understanding how they respond to different therapies [12]. Standard options like chemotherapy and radiotherapy work well for some cancers but can cause side effects such as nausea, hair loss, stomatitis, and leukopenia [17]. Chemotherapy and radiation may also lead to prolonged myelosuppression, reducing granulocytes, increasing infection risk, and weakening the immune system [18,19]. Allogeneic stem cell transplantation is a potential curative option for high-risk or transplant-eligible patients [20]. Ruxolitinib, a *JAK1/JAK2* inhibitor, is commonly used for symptomatic splenomegaly and constitutional symptoms, though *CALR*-mutated patients often have more advanced disease and slightly lower response rates compared to *JAK2*-mutated cases [21,22]. Pegylated interferon-alpha has shown benefits in reducing *CALR* allele burden, making it especially valuable for younger or lower-risk patients [23,24]. These treatments offer potential for deeper molecular remissions and disease modification, though their long-term safety and efficacy are still being studied [24]. Research continues to advance, and promising treatments for *CALR* mutations include monoclonal antibodies, checkpoint inhibitors, and chimeric antigen receptor (CAR) T cell therapy [25,26,27]. This innovative approach modifies a patient’s T cells to better target cancer markers but remains in the early stages for this mutation [27]. The treatment landscape for CALR-mutated post-ET myelofibrosis (MF) is moving toward precision medicine, emphasizing mutation-specific therapies and tailored risk assessments [28]. Targeted therapies for *CALR*-mutation-associated ET with MF represent an exciting area in precision hematologic oncology [29]. Traditional treatments like hydroxyurea, anagrelide, ruxolitinib, and pegylated IFN-α aim to reduce platelet counts and alleviate symptoms but do not directly target the mutant clone [29]. Emerging options, including monoclonal antibodies, bispecific antibodies, and CAR-T therapy, show great promise [29,30].

Recent investigations focus on the mechanisms behind therapy resistance and recurrence, paving the way for more precise and effective treatments to eradicate cancer [31]. Oral tretinoin (all-trans retinoic acid, ATRA), a vitamin A derivative, plays a vital role in HSCs’ biology by interacting with nuclear retinoic acid receptors (RARs) to regulate genes involved in cell differentiation and proliferation [32]. In acute promyelocytic leukemia (APL), tretinoin encourages the maturation of leukemic promyelocytes into granulocytes, reducing malignant cells and restoring normal blood cell production [33]. This has sparked interest in its potential for other HSC-related treatments, such as myelodysplastic syndromes and regenerative medicine, where targeted HSC modulation may offer therapeutic benefits [33]. Immunotherapies have emerged as a promising approach to stimulate tumor-specific immune responses [34]. Cancer immunotherapy has significantly improved patient survival while avoiding the toxicity of traditional chemotherapy or radiotherapy, leading to a better quality of life [26]. Immunotherapy can target CSCs by modulating complex cytokine signaling networks to develop innovative treatments [35]. Cytokines activate effector immune cells to enhance tumor cell recognition by NK cells and cytotoxic T cells [36,37]. Several cytokines, including granulocyte-macrophage-colony-stimulating factor (GM-CSF), IL-7, IL-12, IL-15, and IL-18, have been studied and entered clinical trials [37,38,39]. Two cytokines, interleukin-2 (IL-2) and interferon-alpha (IFN-α), are Food and Drug Administration (FDA)-approved for cancer treatment. IL-2 promotes T cell (CD4+ and CD8+) and NK cell proliferation, enhancing NK cell cytotoxicity [38].

A personalized precision combination approach can integrate various therapies with strategic dosing to minimize toxicity to normal cells while effectively targeting cancer cells. In this report (n of 1), the case study highlights an innovative, personalized precision regimen combining low-dose IL-2 immunotherapy and reduced-dose oral tretinoin targeted therapy, offering care with minimal side effects and maintaining quality of patient life. This method is gaining attention for softening the impact of traditional cancer treatments and enhancing clinical results. Data obtained in this study showed that the patient has achieved disease regression with molecular remission with low-dose personalized precision immunotherapy treatment based on individual immune function profiles and using next-generation sequencing (NGS) of circulating tumor DNA (ctDNA). Table 1 provides a summary of the patient’s demographics and treatment protocols. The patient’s blood counts and symptoms remained stable through monthly cycles of low-dose personalized IL-2 immunotherapy combined with oral tretinoin. No signs of disease progression, such as transformation to myelofibrosis or acute myeloid leukemia (AML), were observed, and molecular remission of *CALR* Type 1 was achieved. The patient maintained a good quality of life with minimal side effects.

## 2. Results

### 2.1. Monitoring White Blood Cells, Red Blood Cells, Hemoglobin, and Platelets in the Peripheral Blood of a Patient Undergoing Low-Dose IL-2 Therapy from March 2020 to September 2025

**Figure 1:**
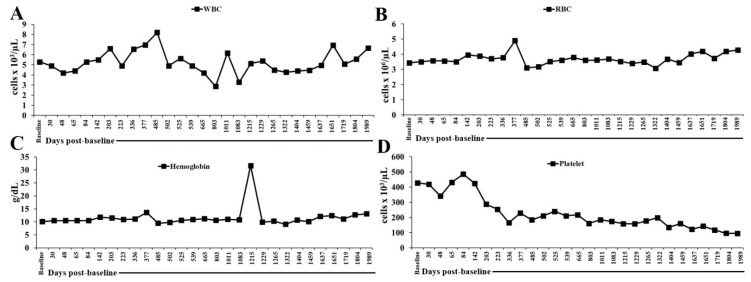
Longitudinal white blood cells, red blood cells, hemoglobin, and platelet count. This plot displays the longitudinal changes in the patient’s (**A**) WBCs, (**B**) RBCs, (**C**) Hb levels, and (**D**) platelet count over a period of nearly four years. The data show fluctuations in these parameters, indicative of various underlying conditions or treatments. This figure provides a comprehensive overview of the patient’s hematological profile during the time frame. The time axis represents days post-first IL-2 administration on 1 July 2020 (baseline or day 0).

In March 2020, blood tests showed normal levels of white blood cells (WBCs) (4.30 × 10^3^/μL), reduced hemoglobin (11.9 g/dL), and normal platelets (223 × 10^3^/μL). On 1 July 2020, the patient received the first dose of IL-2 injections subcutaneously, followed by five administrations per week. IL-2 administration continued throughout the period covered by the data in Figure 1 and Table S1. During IL-2 treatment, the patient’s peripheral blood showed fluctuations in WBC counts, though they remained within the normal range (4.3–10.3 × 10^3^/μL), except on 12 August 2022 (803 days post-baseline analysis) and 19 May 2023 (1083 days post-baseline analysis). Red blood cells (RBCs) were lower than the normal range (4.3–5.7 × 10^6^/μL) except on 13 July 2021 (377 days post-baseline analysis). Hemoglobin (Hb) levels were below the normal range (13.6–17.2 g/dL) except on 13 July 2021 (377 days post-baseline analysis), but remained fairly consistent, aside from significant drops on 29 October 2021 (485 days post-baseline analysis) and 15 November 2021 (502 days post-baseline analysis). Platelet levels were elevated for most of the first five months post-first IL-2 administration but returned to the normal range (159–388 × 10^3^/μL) later, with a notable drop on 5 March 2024 (1404 days post-baseline analysis) (Figure 1 and Table S1).

### 2.2. Flow Cytometry Panel (Innate + Adaptive Immune Profiling) and Monitoring in the Peripheral Blood of a Patient Undergoing Low-Dose IL-2 Therapy

**Figure 2:**
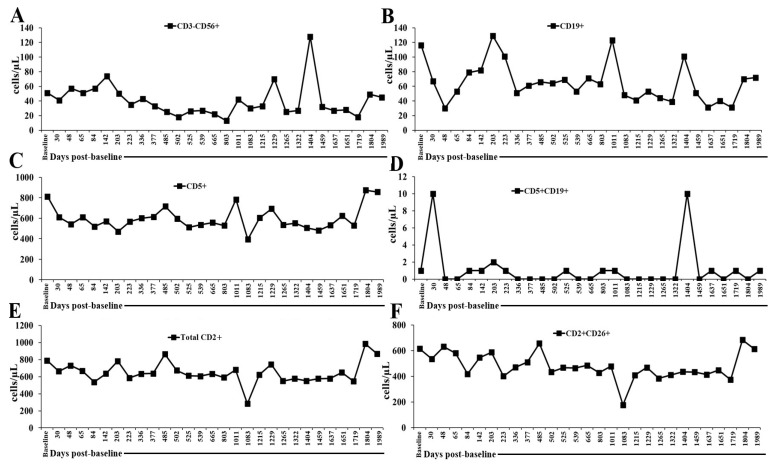
Longitudinal NK cells, T cells, and B cells. (**A**) CD3-CD56+, (**B**) CD19+, (**C**) CD5+, (**D**) CD5+CD19+, (**E**) CD2+, and (**F**) CD2+CD26+ counts were monitored in patient’s peripheral blood at time points as shown in the figure. The time axis represents days post-first IL-2 administration on 1 July 2020 (baseline or day 0).

**Figure 3:**
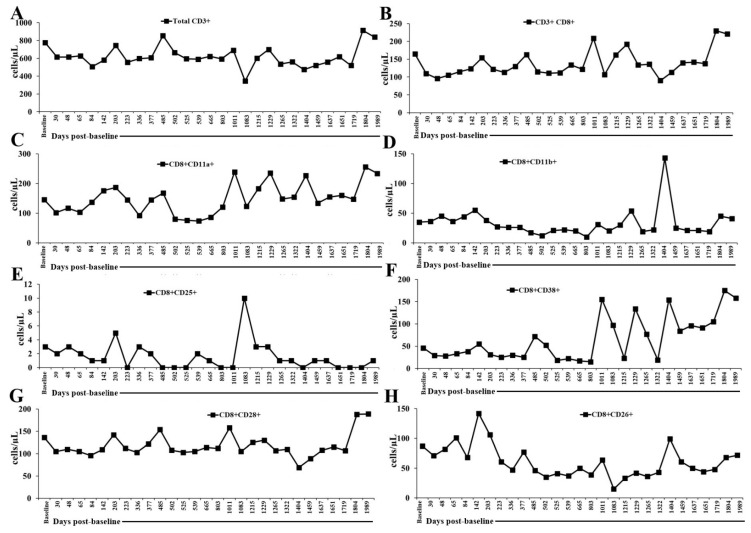
Longitudinal T cells and CD8+T cell subsets. (**A**) CD3+, (**B**) CD3+CD8+, (**C**) CD8+CD11a+, (**D**) CD8+CD11b+, (**E**) CD8+CD25+, (**F**) CD8+CD38+, (**G**) CD8+CD28+, and (**H**) CD8+CD26+ counts were monitored in patient’s peripheral blood at time points as shown in the figure. The time axis represents days post-first IL-2 administration on 1 July 2020 (baseline or day 0).

**Figure 4:**
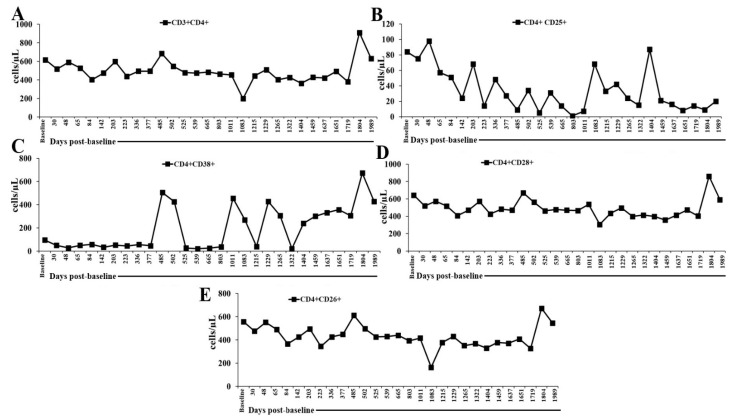
Longitudinal CD4+T cell subsets. (**A**) CD3+CD4+, (**B**) CD4+CD25+, (**C**) CD4+CD38+, (**D**) CD4+CD28+, and (**E**) CD4+CD26+ counts were monitored in patient’s peripheral blood at time points as shown in the figure. The time axis represents days post-first IL-2 administration on 1 July 2020 (baseline or day 0).

**Figure 5:**
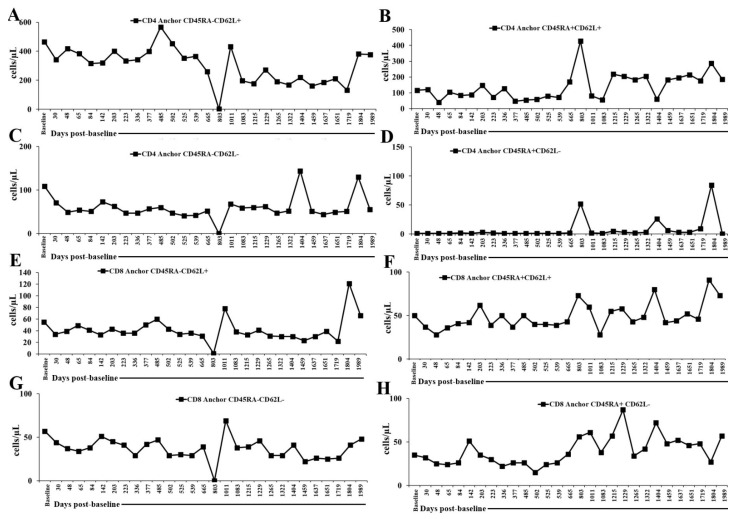
Longitudinal naïve and activated T cell subsets. (**A**) CD4+CD45RA-CD62L+, (**B**) CD4+CD45RA+CD62L+, (**C**) CD4+CD45RA-CD62L-, (**D**) CD4+CD45RA+CD62L-, (**E**) CD8+CD45RA-CD62L+, (**F**) CD8+CD45RA+CD62L+, (**G**) CD8+CD45RA-CD62L-, and (**H**) CD8+CD45RA+CD62L- counts were monitored in patient’s peripheral blood at time points as shown in the figure. The time axis represents days post-first IL-2 administration on 1 July 2020 (baseline or day 0).

CALR-mutant MPNs show impaired function of NK cells, T cells, and B cells driven by cytokine imbalance, chronic inflammation, and stem-cell-derived immunosuppression. Profiling innate and adaptive immune subsets allows for the identification of specific quantitative and functional deficits that underlie disease biology and therapeutic response. These immune parameters were selected to define the specific immune deficits and assess the impact of IL-2 administration throughout the period of immune cell monitoring in the patient’s peripheral blood, with IL-2 treatment maintained throughout this period. Data are shown in Figure 2, Figure 3, Figure 4 and Figure 5 and Tables S2–S5. During IL-2 administration, the patient’s peripheral blood showed fluctuations in immune cell frequencies. NK and B cells were below the normal range (NK: 98–128 cells/μL; B cells: 128–400 cells/μL; CD5+: 1022–1933 cells/μL; CD5+CD19+: 15–178 cells/μL), except that NK cells were within the normal range on 5 March 2024 (1404 days post-baseline analysis). CD2+ cells were within the normal range (CD2: 1130–1985 cells/μL) on 9 April 2025 (1804 days post-baseline analysis), and CD2+CD26+ levels were normal (CD2+CD26+: 593–1375 cells/μL) on 1 July 2020 (baseline), 18 August 2020 (48 days post-baseline analysis), 29 October 2021 (485 days post-baseline analysis), and 9 April 2025 (1804 days post-baseline analysis) (Figure 2 and Table S2). Overall, these findings indicate that continuous IL-2 administration caused a dynamic but selective modulation of peripheral immune cells. The persistently reduced NK cell and B cell counts suggest that low-dose IL-2 primarily expanded regulatory or helper T cell compartments rather than broadly enhancing cytotoxic or antibody-driven (humoral) immunity. In contrast, the stability of CD2+ and CD2+CD26+ T cell populations within normal ranges at multiple time points indicates preserved T cell homeostasis despite fluctuations in other lineages. Clinically, this pattern was consistent with IL-2’s known preferential support of T cell survival and activation—particularly of regulatory and memory phenotypes—while exerting variable effects on NK and B cells. Thus, the immune profile observed during therapy reflects a targeted immunomodulatory response rather than generalized activation.

T cells and their subsets were generally below the normal range, except for CD4+CD26+ cells (normal ranges in cells/μL are as follows for T cells: CD3+: 989–1899; CD3+CD8+: 219–731; CD8+CD11a+: 139–472; CD8+CD11b+: 98–200; CD8+CD25+: 5–50; CD8+CD38+: 50–500; CD8+CD28+: 300–800; CD8+CD26+: 593–1375; CD3+CD4+: 989–1899; CD4+CD25+:10–80; CD4+CD38+: 20–200; CD4+CD28+: 500–1200; CD4+CD26+: 109–1026). CD3+CD8+ T cells were within the normal range only on 5 March 2024 (1404 days post-baseline analysis), while CD4+CD26+ cells remained within the normal range throughout the monitoring period (Figure 3 and Figure 4, and Tables S3 and S4). Taken together, these results show that IL-2 administration was associated with a broad reduction in circulating T cells and most T cell subsets, indicating incomplete or impaired T cell reconstitution during the monitoring period. The consistently low CD8+ populations across activation (CD25+), adhesion (CD11a+/CD11b+), and costimulatory (CD28+) phenotypes suggest limited cytotoxic T cell activation or expansion. In contrast, the persistent normalization of CD4+CD26+ cells indicates selective preservation of a more metabolically active helper T cell subset with known roles in memory formation and cytokine responsiveness. Biologically, this pattern implies that IL-2 therapy may preferentially support certain helper T cell functions while failing to maintain broader T cell subset homeostasis. Clinically, such skewing could reflect a nuanced immune modulation in which helper T cell competence is relatively preserved, whereas cytotoxic T cell-mediated immunity remains constrained and, taken together with the increase in regulatory T cells, prevents the occurrence of an autoimmune effect of the low-dose IL-2.

We also determined the subsets of naïve T cells, CD45RA+ and activated T cells, and the CD45RA- population and found that naïve T cells were mostly lower than the normal range in cells/μL, except CD8+CD45RA+CD62L- T cells. The normal range in cells/μL was as follows: CD4+CD45RA+CD62L+: 280–850; CD4+CD45RA+CD62L-: 10–90; CD8+CD45RA+CD62L+: 140–580; CD8+CD45RA+CD62L-: 10–140. CD8+CD45RA+CD62L- T cells were seen within normal range throughout the monitoring period, whereas CD4+CD45RA+CD62L+ cells were seen in normal range on 12 August 2022 (803 days post-baseline analysis) and 9 April 2025 (1804 days post-baseline analysis), and CD4+CD45RA+CD62L- cells were seen in normal range on 12 August 2022 (803 days post-baseline analysis), 5 March 2024 (1404 days post-baseline analysis), and 9 April 2025 (1804 days post-baseline analysis). Activated T cells were mostly in normal range (CD4+CD45RA-CD62L+: 180–550; CD4+CD45RA-CD62L-: 40–280; CD45RA-CD62L+: 40–380; CD45RA-CD62L-: 15–180), except that CD4+CD45RA-CD62L+ were seen to be low on 12 August 2022 (803 days post-baseline analysis), 29 August 2023 (1215 days post-baseline analysis), 14 December 2023 (1322 days post-baseline analysis), and 14 January 2025 (1719 days post-baseline analysis), CD4+CD45RA-CD62L- and CD8+CD45RA-CD62L-cells were seen to be low on 12 August 2022 (803 days post-baseline analysis) (Figure 5 and Table S5). The naïve and activated T cell profiles indicate a skewed and selectively preserved pattern of T cell homeostasis during IL-2 administration. The consistent reduction in most naïve T cell subsets, particularly CD4+CD45RA+ populations, suggests impaired replenishment or reduced survival of the naïve T cell pool. This pattern is commonly associated with chronic immune activation, reduced thymic output, or selective cytokine-driven maturation pressures, all of which can be seen in settings where IL-2 signaling favors expansion or survival of more differentiated T cell phenotypes. In contrast, the stable levels of CD8+CD45RA+CD62L+ cells across the entire monitoring period point to preserved or preferential maintenance of this subset, which includes terminally differentiated effector memory RA-positive (TEMRA-like) cells. This preservation may reflect heightened IL-2 sensitivity or increased homeostatic turnover of cytotoxic effector-type CD8+ cells. Activated T cell subsets remained largely within normal range, indicating that, despite reduced naïve cell availability, the patient maintained functional activation capacity among both CD4+ and CD8+ T cells. The occasional decreases observed at specific time points likely reflect transient immune variation rather than sustained dysfunction. Clinically, this immune signature points to an immune system that is capable of activation and effector responses but constrained in its ability to generate or sustain a robust naïve T cell reservoir. This imbalance could influence long-term immune adaptability and preserve immediate effector potential while potentially limiting the breadth of responses to new antigens.

### 2.3. Monitoring Cytotoxic Activity of NK Cells and Cytokine Release by the Plasma of a Patient Undergoing Low-Dose IL-2 Therapy

**Figure 6:**
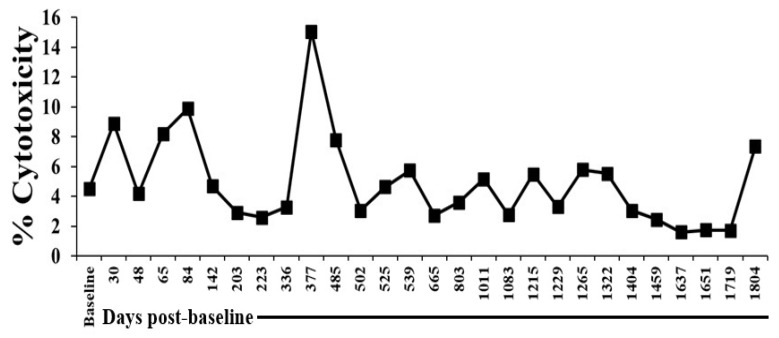
Longitudinal cytotoxic activity of NK cells. Cytotoxic activity of PBMCs was determined against K562 (chronic myelogenous leukemia) using a ^51^chromium-release assay. CD56+CD3- NK cell percentages in PBMCs were determined by flow cytometry; an effector: target ratio of 1:1 (CD56+CD3-: K562) was used. The time axis represents days post-first IL-2 administration on 1 July 2020 (baseline or day 0).

The observed fluctuations in NK cell cytotoxic activity, characterized by an initial peak on 13 July 2021 (377 days post-baseline analysis) and followed by a later restoration on 9 April 2025 (1804 days post-baseline analysis), demonstrate a dynamic pattern of immune reactivation during low-dose IL-2 therapy. Importantly, the full restoration of NK cell function coincided with the time point at which the Type 1 *CALR* mutation became undetectable, suggesting that elimination of the *CALR*-mutant clone relieved an underlying immunosuppressive pressure on NK cells (Figure 6). Monitoring cytokine release in the plasma of this patient undergoing IL-2 therapy showed intermittent elevations of several pro-inflammatory cytokines, including IFNγ, TNFα, IL1α/β, IL6, IL12, and IL2, often exceeding the normal healthy ranges (IFNγ: 0–20; TNFα: 0–15; Ltα (TNFβ): 0–5; TNF RI: 550–950; TNF RII: 350–750; IL1α: 0–5; IL1β: 0–5; IL6: 0–7; IL12: 0–5; IL2: 0–5; IL15: 0–2.5; IL8: 0–10; IL4: 0–5; IL5: 0–5; IL17: 0–3; IL23: 0–3; IL10: 0–8; IL13: 0–4). These cytokine surges are consistent with periods of heightened immune activation driven by IL-2 therapy, reflecting stimulation of both innate and adaptive immune pathways. The recurrent elevation of NK and T cells activating cytokines (IL-2, IL-15, IFNγ, TNFα) indicates that IL-2 was not simply expanding immune cell counts but actively reenergizing functional immune responses (Table 2).

### 2.4. Symptoms and Health Monitoring of a Patient Undergoing Low-Dose IL-2 Therapy

The patient continued cycles of low-dose personalized IL-2 immunotherapy with oral tretinoin, which improved symptoms of fatigue and splenomegaly without evidence of myelofibrosis progression. By January 2025 (1700+ days post-baseline analysis), further analysis showed normalized plasma cytokine levels, including TNF-RII at 350 pg/mL, IFN-γ at 10 pg/mL, and IL-4 at 3 pg/mL. TNF-RII and IFN-γ are involved in antitumor immunity, while IL-4 is associated with an anti-inflammatory response (Table 2). Therefore, this trend indicates a favorable immune response and potential control of tumor growth. Overall, the treatment appears to have effectively managed the patient’s condition, with promising signs of improved immune surveillance and cancer control.

### 2.5. CALR Mutation Monitoring in a Patient Undergoing Low-Dose IL-2 Therapy

**Figure 7:**
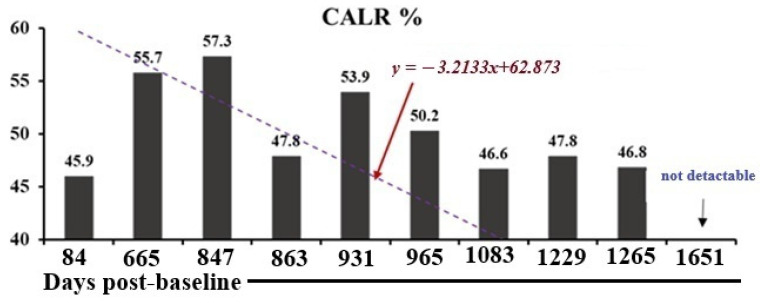
CALR mutation percentage decline. Graph of CALR-mutant allele burden (%) at each sampling point. A downward trend is apparent, and the dotted line represents the linear-regression best fit (*y = mx + b*). The decline suggests a favorable molecular response to the therapy being administered. The time axis represents days post-first IL-2 administration on 1 July 2020 (baseline or day 0).

In November 2024 (1651 days post-baseline analysis), molecular pathology revealed that the Type 1 *CALR* mutation was no longer detected, with the emergence of senescent biomarkers instead (Figure 7). This is a significant finding, as the calreticulin mutation strongly indicates a myeloid neoplasm and serves as a marker for disease progression. The Type 1 CALR mutation has remained undetectable.

## 3. Discussion

When normal stem cells mutate to cancer-initiating stem cells, these CSCs become key players in cancer initiation and progression. Despite significant research into pathways, tumor suppressor genes, oncogenes, and mutations that increase tumor burden, current treatment options are still lacking. Chemotherapy and radiation therapy are the usual standards for addressing CSCs in solid tumors and blood cancers, but these treatments often come with severe side effects, toxicities, and a reduced quality of life. Additionally, resistance to traditional therapies frequently leads to high recurrence rates. A promising alternative is personalized precision treatment plans that combine low-dose immunotherapy with reduced-dose targeted therapy, specifically aiming at CSCs while minimizing harmful side effects.

We present a case in which the patient achieved molecular remission of the MPN-associated Type 1 *CALR* mutation using a personalized low-dose immunotherapy approach, combining rIL-2 with oral tretinoin targeted therapy. Patients with Type 1 *CALR* mutations experience a more aggressive disease course with rapid progression to AML as compared to individuals with Type 2 mutations. We precisely adjusted the low-dose rIL-2 regimen for this patient based on immune effector cell levels in peripheral blood before and after treatment cycles. The goal was to mobilize and activate functional bone marrow stem cells (BMSCs) and enhance immune cells, particularly NK cell function, to increase their cytotoxic and cytokine activity against cancer stem cells. We present further evidence of the efficacy of low-dose IL-2 with oral tretinoin in two additional cases, showing that the improvement in NK cell function resulted in the achievement of hematological remission. In case 2 with an MPN, specifically *JAK2*-positive ET, the patient achieved hematologic remission with one cycle of treatment. Patient was a 57-year-old man with *JAK2*-positive ET who received daily low-dose subcutaneous IL-2 (10–20,000 IU/kg, five days a week) plus reduced-dose tretinoin, with dosing and frequency tailored to immune panel results and disease monitored through blood counts, flow cytometry, and molecular analysis. By day 16, NK cell count rose to 397 cells/µL with normal function at 21.32%, alongside improved CD8+ and CD4+ T cell counts and normalized platelets. However, this remission was not sustained, indicating that multiple cycles of treatment are necessary to maintain NK cell function for activity against cancer stem cell-driver mutations, in this case, JAK2. Case 3 was a 62-year-old woman negative for *JAK2*, *CALR*, and MPL mutations who was treated similarly with IL-2 and tretinoin. After five cycles, she achieved normal NK and T cell function, hematologic remission, no detectable driver mutations, and no molecular evidence of epigenetic or spliceosome gene mutations such as *TET2*, *ASXL1*, *SRSF2*, *EZH2*, *SETD2*, and *SF3B1*.

NK cells are cytotoxic lymphocytes, and tumor cells often lack or have reduced major histocompatibility complex (MHC)-I expression, making them vulnerable to NK cell attacks [40]. Tumor cells may also express ligands that interact with NK cells’ activating receptors and trigger NK cells’ functional killing and enhance their therapeutic potential in cancer treatment [40]. IL-2, a cytokine, promotes NK cell development, and low-dose IL-2 infusions have been shown to increase NK cell activity, enhance cytotoxicity, and reduce tumor burden [41].

Our treatment involved cycles of rIL-2 and oral tretinoin with periodic breaks to minimize side effects. After two cycles, the patient responded well, but after five more, NK cell function declined, leading to a treatment pause. The patient then received alpha-interferon before resuming rIL-2 therapy. This strategy combined IL-2’s stimulation of NK cells with alpha-interferon’s activation of B cells, T cells, and NK cells. Interferon also induced a molecular response in patients with *JAK2V617F* and *CALR* mutations by activating dormant stem cell populations [42]. Following repeated rIL-2 cycles based on immune measurements, the patient showed increased NK cell counts, and molecular analysis indicated no detectable *CALR* mutation. CALR supports MHC class I molecule connections, enhances leukocyte tumor infiltration, and strengthens immunotherapy’s antitumor effects. *CALR* mutation might significantly affect IL-2 immunotherapy’s long-term outcomes. This therapy targets bulk tumor cells and CSCs, with IL-2 promoting TRAIL and granulysin expression on NK cells to drive apoptosis in CSCs, regardless of antigen heterogeneity. IL-2 signaling also temporarily drives CSCs out of quiescence, making them vulnerable to immune attack. Combining oral tretinoin further reduced stemness markers (e.g., CD133, ALDH1) and re-sensitized CSCs to NK cell cytotoxicity [43,44]. Low-dose IL-2 not only expands effector populations but also creates a niche where CSCs are targeted and eradicated, achieving lasting molecular remission [45,46].

The targeted immunotherapy provided to the patient was customized to address specific immune dysfunctions. Low-dose rIL-2 served as a key cytokine, enhancing CD4+ CD8+ T cell and NK cell cytotoxicity while maintaining minimal toxicity and effectively controlling disease progression. Combining IL-2 with low-dose targeted therapies boosted antineoplastic activity. For example, the IL-2 and alpha interferon combination sustained immune activation, with alpha interferon’s platelet-lowering effect particularly aiding patients with thrombocytosis. Adhering to treatment protocols consistently improved functional NK cell cytotoxicity and counts. Oral tretinoin supported IL-2-mediated T cell proliferation. Other targeted therapies with immunotherapy include Revlimid with IL-2, which has been highly effective for lymphomas and leukemias, as Revlimid induced CD4+ T cell secretion of IL-2 and stimulated NK cells [47]. When paired with granulocyte-colony-stimulating factor (G-CSF), IL-2 increased IL-2 receptor expression on T lymphocytes through dendritic cell and macrophage priming [48]. Importantly, in this report, the patient experienced minimal side effects during treatment cycles with low-dose recombinant human (rh)IL-2 and oral tretinoin.

Overall, these results highlight the exciting potential of personalized precision immunotherapy combination treatments to help the innate immune system restore its ability to target cancer cells in patients with Type 1 *CALR* mutations with a more aggressive disease course (Figure 6). In addition, our report highlights the importance of patient preference and choice in the participation of their care and consideration of all treatment options available, with emphasis on maintaining patients’ quality of life while choosing outpatient treatments with minimal side effects and achieving good outcomes.

## 4. Conclusions

Immunotherapy has been a reliable treatment for years, but ongoing advancements, as presented in this report of personalized precision regimens, are boosting its potential as a top option. Its key benefit lies in its lower toxicity and fewer side effects compared to traditional methods. Our research highlights the success of combining IL-2 with targeted therapy for both solid tumors and blood cancers. The treatment plan aimed to optimize NK cell count, correct immune dysfunction, and achieve molecular remission. Impressively, the patient tolerated multiple cycles without issues. After two treatment cycles, NK cell function improved, but further cycles caused a decline, emphasizing the importance of personalized, cancer-specific dosing. Factors like the patient’s *CALR* mutation could impact IL-2-induced antitumor responses. By September 2025 (1989 days post-baseline analysis), the patient noted a 90% reduction in symptoms compared to earlier evaluations. Immune panel results showed that the highest level of NK cell count achieved was normal at 128 cells/μL. Future studies should investigate cytokine signaling pathways to enhance NK cell activation. As research evolves, immunotherapy targeting these pathways is expected to gain more attention in cancer care.

## 5. Materials and Methods

### 5.1. Case Presentation

A 65-year-old female patient presented to The Maharaj Institute in March 2020 with a myeloproliferative neoplasm (MPN), specifically, post-essential thrombocythemia myelofibrosis (ETMF) with a Type 1 CALR mutation.

She was first diagnosed with ET in 1996 and treated with hydroxyurea, initially 500 mg daily, then 1000 mg every third day from 1998 to 2004, which normalized her platelet count. In 2004, she discontinued hydroxyurea and opted for alternative medicine, including supplements, bowel cleanses, and plant-based foods. Her platelet count remained stable until 2015, when the disease progressed, accompanied by rising platelet counts. A September 2015 bone marrow biopsy revealed MPN classified as post-ETMF with severe reticulin fibrosis (WHO grade 3+/3). This was associated with advanced disease, worsened hematopoiesis, cytopenia, and a higher risk of progression to acute leukemia. Molecular studies confirmed a Type 1 *CALR* mutation but no JAK2 or MPL mutations. Hydroxyurea was restarted at 500 mg daily and later adjusted to 1000 mg every third day, stabilizing her platelet count, though it remained mildly elevated. A bone marrow transplant was considered as a treatment option.

In January 2017, she developed autoimmune Guillain–Barré syndrome, leading to an 8-day paralysis. She was successfully treated with plasmapheresis, steroids, and a 6-week hospitalization. Residual complications included persistent right-shoulder pain and peripheral neuropathy. In November 2019, further studies showed normal von Willebrand factor (VWF) distribution and a 52 bp deletion in *CALR* exon 9, with *JAK2 V617F* remaining negative. By February 2020, her condition was stable, and a bone marrow transplant was again considered as a treatment option.

In March 2020, the patient visited our institute with fatigue and an enlarged spleen. Blood tests revealed normal levels of white blood cells (4.30 × 10^3^/μL), hemoglobin (11.9 g/dL), and platelets (223 × 10^3^). Monitoring was focused on minimizing thrombocytosis to lower the risk of blood clots. Regular check-ups on symptoms, spleen size, blood counts, and supportive care were conducted, including managing anemia and fatigue to adjust treatment as needed.

Tests showed immune dysfunction, with low natural killer cells and reduced natural killer cell function in innate immunity. Adaptive immunity showed decreased levels of T helper cells, T cytotoxic cells, total T cells, regulatory T cells, and naïve T cells, indicating immune aging. Treatment discussions included options of standard care, no treatment, bone marrow transplantation, or addressing immune deficiencies with experimental low-dose personalized IL-2 immunotherapy combined with reduced-dose oral tretinoin and post-treatment monitoring. The patient acknowledged the lack of guaranteed improvement and considered alternatives, including bone marrow transplantation. Potential side effects of low-dose IL-2, such as injection-site reactions and mild flu-like symptoms, manageable with over-the-counter medication, were also explained.

In June 2020, the patient provided informed consent and agreed to the publication of the results for low-dose personalized IL-2 immunotherapy with reduced-dose oral tretinoin, aimed at selectively improving immune dysregulation. The treatment involved cycles of daily low-dose subcutaneous IL-2 injections at 20,000 IU/kg, administered five days a week from 1 July 2020 until 11 September 2025. Adjustments to the duration, dosing, and frequency of IL-2 cycles were guided by peripheral blood immune panel results. Disease progression was monitored through peripheral blood counts, flow cytometry, and molecular analysis.

By November 2020, the patient’s clinical status and blood counts remained stable, with mild anemia but no additional cytopenia, thrombosis, worsening myelofibrosis, or acute leukemia. Flow cytometry revealed an abnormal B cell population and a mild left-shifted maturation pattern with 0.2% myeloblasts. Molecular pathology confirmed the persistence of a Type 1 *CALR* mutation, with *JAK2 V617F* being negative.

### 5.2. Surface Staining

Staining was performed by labeling the cells with antibodies as described previously [49,50,51]. Flow-cytometric analysis was performed using an AttuneTM NxT flow cytometer (Thermo Fisher Scientific, Waltham, MA, USA), and the results were analyzed by the FlowJo v10 software (BD Biosciences, Ashland, OR, USA).

### 5.3. ^51^Chromium Release Cytotoxicity Assay

A ^51^chromium (^51^Cr) release assay was performed as described previously [52]. Briefly, different numbers of effector cells were incubated with ^51^Cr-labeled target cells. After a 4 h incubation period, the supernatants were harvested from each sample, and the released radioactivity was counted using a gamma counter. The percentage specific cytotoxicity was calculated as follows:

$$\begin{array}{ll} \%\;\mathrm{Cytotoxicity}\;=\; & \underline{\mathrm{Experimental}\;\mathrm{cpm}\;-\;\mathrm{spontaneous}\;\mathrm{cpm}\;}\\ & \;\;\mathrm{Total}\;\mathrm{cpm}\;-\;\mathrm{spontaneous}\;\mathrm{cpm} \end{array}$$

Lytic units (LUs) 30/10^6^ were calculated by using the inverse of the number of effector cells needed to lyse 30% of tumor target cells × 100.

### 5.4. Enzyme-Linked Immunosorbent Assays (ELISAs)

ELISAs were performed as previously described [51]. To analyze and determine the concentration of cytokines and chemokines, a standard curve was generated using either two- or three-fold dilutions of recombinant cytokines provided by the manufacturer.

## Figures and Tables

**Table 1:** Patient demographics and treatment summary.

Diagnosis/Mutation	Age/Sex	Previous Treatment/Cycles	Lowest/Highest NK Cell Counts	Lowest/Highest NK Cell Cytotoxicity	Molecular Remission After IL-2 Treatment	Targeted Therapy	Time Since Molecular Remission (Months)	Survival Since Diagnosis (Months/Years)	Toxicity of Low-Dose Targeted Therapy and Immune-Therapy (Grade 0–4)
ETMF/ CALR	65/ Female	Holistic hydroxy-urea/42	13/128 cells/µL	1.60/15.8%	CALR absent	Retinoic acid, alpha interferon	11	444/37	1

Table 1 Patient demographics, treatment history, and clinical outcomes. This table summarizes key clinical and therapeutic parameters for a patient with post-essential thrombocythemia myelofibrosis (ETMF) treated with a personalized low-dose IL-2 immunotherapy regimen combined with targeted therapy. Data include diagnosis, age, sex, prior treatments, number of IL-2 cycles administered, lowest and highest NK cell counts (normal range: 98–128 cells/µL), lowest and highest NK cell cytotoxicity (normal range: 16.3–39.9%), mutation status, achievement of molecular remission, targeted therapy used, time since molecular remission, overall survival since diagnosis, and toxicity grade (0–4) for low-dose targeted therapy and immunotherapy.

**Table 2:** Monitoring of cytokine and chemokine release in peripheral-blood-derived plasma. (A,B) Factors shown in the table were monitored in patient’s peripheral-blood-derived plasma at the time points shown in the table.

**(A) Factors released in the peripheral-blood-derived plasma**									
**Days Post-Baseline**	**IFNγ** **Normal: 0–20**	**TNFα** **Normal: 0–15**	**Ltα(TNFβ) Normal: 0–5**	**TNF RI** **Normal: 550–950**	**TNF RII Normal:** **350–750**	**IL1α** **Normal: 0–5**	**IL1β** **Normal: 0–5**	**IL6** **Normal: 0–7**	**IL12** **Normal: 0–5**
Baseline	5.98	7.16	23.18	574.51	687.05	12.02	16.94	6.82	3.95
30 days	6.56	3.39	11.01	516.52	560.05	7.41	14.56	0.75	1.38
48 days	5.75	3.51	6.55	602.29	762.28	6.26	12.3	2.13	0.92
65 days	8.01	5.75	12.17	645.61	671.62	4.47	8.1	3.12	BLD
84 days	7.07	0.52	16.28	459.54	570.27	6.34	8.7	1.2	BLD
142 days	13.33	7.44	6.86	505.66	590.91	3.39	27.91	4.76	BLD
203 days	7.2	4.99	11.27	574.49	805.6	10.07	13.04	8.1	2.71
485 days	5.04	1.05	BLD	1160.16	925.85	9.1	8.24	2.4	3.23
502 days	2.89	13.72	5.17	1771.17	1362.28	17.59	13.32	6.78	5.02
525 days	23.26	36.54	0.14	1195.64	944.33	11.89	9.79	5.15	5.25
803 days	2.59	11.16	2.37	607.63	783.37	6.56	6.77	3.05	2.52
1011 days	6.94	10.82	7.47	591.68	734.93	22.54	20.97	7.3	2.9
1083 days	4.84	7.03	4.21	616.24	830.71	4.03	6.47	3.36	1.57
1265 days	5	9.41	1.7	585.93	900.86	4.64	2.7	2.42	1.02
1322 days	2.54	6.94	5.52	687.11	1012.1	3.18	4.59	3.19	1.37
1404 days	3.54	12.29	3.31	885.53	1004.34	4.21	7.87	4.99	1.46
1459 days	4.8	3.46	5.02	794.61	965.04	4.39	5.01	1.37	0.63
1637 days	4.97	11.2	4.52	878.19	931.83	4.05	7.39	4.8	2.25
1651 days	4.4	6.05	2.92	825.25	947.36	3.15	6.96	3.12	2.23
1719 days	6.11	6.57	5.21	586.35	673.47	2.54	5.57	10.1	1.88
1804 days	4.6	4.3	4.6	554.4	645.8	3.6	4.2	4.4	2.2
**(B) Factors released in the peripheral-blood-derived plasma**									
**Days Post-Baseline**	**IL2** **Normal:** **0–5**	**IL15 Normal:** **0–2.5**	**IL8 Normal:** **0–10**	**IL4 Normal:** **0–5**	**IL5** **Normal:** **0–5**	**IL17 Normal: 0–3**	**IL23** **Normal: 0–3**	**IL10** **Normal:** **0–8**	**IL13** **Normal: 0–4**
Baseline	6.38	1.56	1.85	0.96	3.27	10.97	57.99	5.78	2.9
30 days	6.07	0.92	1.34	1.51	4.37	7.65	20.76	13.65	2.47
48 days	10.85	1.67	0.98	0.33	2.46	5.28	40.29	9.5	3.67
65 days	7.87	1.73	1.02	3.03	2.21	3.25	16.89	13.81	2.61
84 days	11.51	1.47	<0.25	1.77	1.65	3.26	14.36	12.47	4.19
142 days	9.62	2.17	1.22	3.46	4.63	6.34	19.62	2	4.47
203 days	9.39	7.29	1.23	3.87	2.7	22.34	13.03	16.02	4.88
485 days	28.04	10.52	1.34	0.2	1.81	BLD	79.57	BLD	2.55
502 days	23.52	31.72	2.47	2.63	5.08	BLD	BLD	36.04	1.95
525 days	16.42	17.86	1.57	0.96	2.48	77.21	184.76	24.85	3.98
803 days	3.75	2.48	3.35	0.95	3.84	6.67	15.13	15.34	4.8
1011 days	10.26	3.15	3.75	1.9	4.27	7.47	55.4	14.58	4.72
1083 days	5.36	2.47	3.47	0.56	3.32	3.26	13.49	3.16	1.98
1265 days	4.77	4.74	6.49	0.26	2.36	0.14	9.78	8.86	1.49
1322 days	3.82	2.02	3.8	0.54	0.93	4.24	10.25	3.64	1.19
1404 days	4.63	4.6	4.87	0.98	1.87	6.36	16.01	16.22	1.96
1459 days	6.03	5.11	3.29	0.47	2.35	2.87	16.56	9.43	1.87
1637 days	7.98	4.6	4.53	0.84	1.6	6.05	13.08	6.67	1.96
1651 days	7.98	2.48	2.17	0.49	1.59	4.97	12.93	13.92	1.98
1719 days	5.72	2.84	4.34	0.52	1.75	3.92	21.89	8.01	1.24
1804 days	6.1	2.3	7	1.9	1.1	6	20.1	4.8	1.72

## Data Availability

The original contributions presented in this study are included in the article/Supplementary Materials. Further inquiries can be directed to the corresponding author.

## Supplementary Materials

The following supporting information can be downloaded at: https://www.mdpi.com/article/10.3390/ijms27062814/s1.

## Abbreviations

The following abbreviations are used in this manuscript:

MPN	Myeloproliferative neoplasm
LT-HSCs	Long-term hematopoietic stem cells
NK	Natural killer
CSCs	Cancer stem cells
IFN-γ	Interferon-gamma
TNF-α	Tumor necrosis factor-alpha
CALR	Calreticulin
SCs	Stem cells
MPL	Myeloproliferative leukemia
JAK-STAT	Janus Kinase-Signal Transducer and Activator of Transcription
ET	Essential thrombocytopenia
MF	Myelofibrosis
IFN-α	Interferon-alpha
IL	Interleukin
AML	Acute myeloid leukemia
CD	Cluster of differentiation
