# Supplementary material for: Interleukin-2 and Tretinoin for Myeloproliferative Neoplasms and to Target Type 1 Calreticulin-Driven Neoplasms: Advancements in Immune Regenerative Medicine
Source: Int J Mol Sci. 2026 Mar 20;27(6):2814. doi: 10.3390/ijms27062814 (PMC13027028; doi:10.3390/ijms27062814)
Supplement: Supplementary file 1 [file ijms-27-02814-s001.zip › ijms-4091858-supplementary.pdf]

Supplementary Data

Table S1. Longitudinal white blood cells, red blood cells, hemoglobin, and platelet count. This Table provides data on patient's white blood cells (WBCs), red blood cells (RBCs), hemoglobin levels, and platelet count over a period of nearly four years.

Month.day.year	WBCs<br>*Normal: 4.3-10.3×10^3/μL*	RBCs<br>*Normal: 4.3-5.7 x10^6/μL*	Hemoglobin<br>*Normal: 13.6-17.2 g/dL*	Platelets<br>*Normal: 159-388×10^3/μL*
7.1.20	5.3	3.44	10.1	428
7.31.2020	4.9	3.51	10.5	419
8.18.2020	4.2	3.58	10.5	341
9.4.2020	4.4	3.56	10.5	432
9.23.2020	5.3	3.51	10.5	486
11.20.2020	5.5	3.96	11.8	425
1.20.2021	6.6	3.88	11.5	288
2.9.2021	4.9	3.71	10.9	254
6.2.2021	6.6	3.78	11.1	166
7.13.2021	7.0	4.90	13.6	229
10.29.2021	8.2	3.11	9.5	184
11.15.2021	4.9	3.17	9.8	211
12.8.2021	5.6	3.52	10.6	240
12.22.2021	4.9	3.61	10.9	211
4.27.2022	4.2	3.79	11.2	218
8.12.2022	2.9	3.60	10.6	161
3.8.2023	6.2	3.62	11.0	185
5.19.2023	3.3	3.69	10.8	174
8.29.2023	5.1	3.53	31.7	159
9.12.2023	5.4	3.40	9.9	158
10.18.2023	4.5	3.49	10.3	177
12.14.2023	4.3	3.08	9.1	199
3.5.2024	4.4	3.68	10.7	135
4.29.2024	4.5	3.45	10.1	160
10.24.2024	5.0	4.03	12.1	122
11.7.2024	7.0	4.19	12.4	143
1.14.2025	5.1	3.73	11.1	118
4.9.2025	5.6	4.19	12.7	96
9.10.2025	6.65	4.28	13.1	95.0

Table S2. Longitudinal NK cells, T cells, and B cells. CD3-CD56+, CD19+, CD5+, CD2+, CD5+CD19+, and CD2+CD26+ counts were monitored in patient's peripheral blood at time points as shown in the Table.

Month.day.year	NK cells *Normal: 98-128*	B cells *Normal: 128-400*	CD5+ *Normal: 1022-1933*	CD5+CD19+ *Normal: 15-178*	CD2+ *Normal:1130-1985*	CD2+CD26+ *Normal: 593-1375*
7.1.20	51	116	813	1	789	615
7.31.2020	41	67	610	10	663	536
8.18.2020	57	30	542	0	731	631
9.4.2020	51	53	612	0	667	581
9.23.2020	57	79	520	1	537	418
11.20.2020	74	82	572	1	636	546
1.20.2021	50	129	471	2	782	588
2.9.2021	35	101	569	1	585	401
6.2.2021	43	51	601	0	633	470
7.13.2021	33	61	614	0	637	511
10.29.2021	25	66	717	0	863	656
11.15.2021	18	64	597	0	673	434
12.8.2021	26	69	513	1	612	468
12.22.2021	27	53	537	0	606	464
4.27.2022	22	71	559	0	633	485
8.12.2022	13	63	530	1	591	428
3.8.2023	42	123	784	1	680	478
5.19.2023	30	48	395	0	284	177
8.29.2023	33	41	604	0	622	409
9.12.2023	70	53	694	0	744	469
10.18.2023	25	44	537	0	550	384
12.14.2023	27	39	553	0	577	411
3.5.2024	128	101	507	10	551	436
4.29.2024	32	51	482	0	577	434
10.24.2024	27	31	532	1	579	414
11.7.2024	28	40	624	0	650	448
1.14.2025	18	31	529	1	545	374
4.9.2025	49	70	875	0	984	684
9.10.2025	45	72	858	1	866	613

Table S3. Longitudinal T cells and CD8+T cell subsets. CD3+, CD3+CD8+, CD8+CD11a+, CD8+CD11b+, CD8+CD25+, CD8+CD38+, CD8+CD28+, and CD8+CD26+ counts were monitored in patient's peripheral blood at time points shown in the Table.

**Table S3** Month.day.year	T cells *Normal: 989-1899*	CD3+ CD8+ *Normal: 219-731*	CD8+CD11a+ *Normal: 139-472*	CD8+CD11b+ *Normal: 98-200*	CD8+CD25+ *Normal: 5-50*	CD8+CD38+ *Normal: 50-500*	CD8+CD28+ *Normal: 300-800*	CD8+CD26+ *Normal: 593-1375*
7.1.20	776	165	146	35	3	46	136	87
7.31.2020	616	110	102	36	2	29	105	71
8.18.2020	615	96	117	45	3	28	110	82
9.4.2020	629	105	103	36	2	33	105	101
9.23.2020	508	115	137	44	1	38	96	68
11.20.2020	581	123	176	55	1	55	109	142
1.20.2021	745	154	187	38	5	31	142	106
2.9.2021	556	122	145	27	0	25	112	61
6.2.2021	598	113	92	26	3	30	103	47
7.13.2021	607	130	145	26	2	25	122	77
10.29.2021	855	163	168	17	0	72	154	46
11.15.2021	665	115	80	12	0	52	108	35
12.8.2021	596	111	76	21	0	18	103	41
12.22.2021	591	112	74	22	2	22	105	37
4.27.2022	623	134	86	20	1	17	114	50
8.12.2022	594	122	121	10	0	15	112	39
3.8.2023	691	209	239	31	0	155	158	64
5.19.2023	345	107	123	20	10	97	105	15
8.29.2023	601	162	183	30	3	23	125	33
9.12.2023	700	192	235	54	3	134	130	42
10.18.2023	537	134	148	19	1	77	107	36
12.14.2023	561	136	154	22	1	19	110	43
3.5.2024	475	90	227	143	0	154	69	99
4.29.2024	520	113	134	25	1	84	89	61
10.24.2024	558	140	155	21	1	96	108	50
11.7.2024	619	142	160	21	0	91	115	44
1.14.2025	520	138	147	19	0	105	107	48
4.9.2025	914	230	256	45	0	175	188	68
9.10.2025	839	221	234	41	1	158	189	72

Table S4. Longitudinal CD4+T cell subsets. CD3+CD4+, CD4+CD25+, CD4+CD38+, CD4+CD28+, and CD4+CD26+ counts were monitored in patient's peripheral blood at time points shown in the Table.

Month.day.year	CD3+ CD4+ *Normal: 989-1899*	CD4+CD25+ *Normal: 10-80*	CD4+CD38+ *Normal: 20-200*	CD4+CD28+ *Normal: 500-1200*	CD4+CD26+ *Normal: 109-1026*
7.1.20	617	84	96	642	557
7.31.2020	518	75	50	518	476
8.18.2020	591	98	28	572	552
9.4.2020	528	57	50	517	491
9.23.2020	403	51	58	407	367
11.20.2020	474	24	34	469	425
1.20.2021	598	68	52	571	495
2.9.2021	438	14	45	425	345
6.2.2021	495	48	57	482	427
7.13.2021	495	27	46	469	448
10.29.2021	684	9	505	670	612
11.15.2021	547	34	426	562	497
12.8.2021	479	5	27	462	427
12.22.2021	474	31	21	477	430
4.27.2022	485	14	26	470	440
8.12.2022	464	1	37	465	393
3.8.2023	454	7	456	537	416
5.19.2023	199	68	270	306	164
8.29.2023	445	33	40	435	378
9.12.2023	510	42	427	496	430
10.18.2023	403	24	306	397	352
12.14.2023	427	15	21	413	368
3.5.2024	364	87	240	397	330
4.29.2024	429	21	301	357	378
10.24.2024	421	16	332	411	371
11.7.2024	492	8	357	473	408
1.14.2025	381	14	305	402	326
4.9.2025	910	9	673	860	673
9.10.2025	631	20	427	591	545

Table S5. Longitudinal naïve and activated T cell subsets. CD4+CD45RA-CD62L+, CD4+CD45RA+CD62L+, CD4+CD45RA-CD62L-, CD4+CD45RA+CD62L-, CD8+CD45RA-CD62L+, CD8+CD45RA+CD62L+, CD8+CD45RA-CD62L-, and CD8+CD45RA+CD62L- counts were monitored in patient's peripheral blood at time points shown in the Table.

**Table S5** Month.day.year	CD4 Anchor CD45RA-CD62L+ *Normal: 180-550*	CD4 Anchor CD45RA+CD62L+ *Normal: 280-850*	CD4 Anchor CD45RA-CD62L- *Normal: 40-280*	CD4 Anchor CD45RA+CD62L- *Normal: 10-90*	CD8 Anchor CD45RA-CD62L+ *Normal: 40-380*	CD8 Anchor CD45RA+CD62L+ *Normal: 140-580*	CD8 Anchor CD45RA-CD62L- *Normal: 15-180*	CD8 Anchor CD45RA+CD62L- *Normal: 10-140*
7.1.20	465	116	109	1	55	50	57	35
7.31.2020	343	121	71	1	34	37	44	32
8.18.2020	418	39	49	1	39	28	37	25
9.4.2020	383	104	54	1	49	36	34	24
9.23.2020	316	84	51	2	41	41	38	26
11.20.2020	320	88	73	1	33	42	51	51
1.20.2021	400	147	63	3	43	62	45	35
2.9.2021	333	72	47	2	36	39	41	30
6.2.2021	342	127	47	1	36	50	29	22
7.13.2021	399	47	57	1	50	37	42	26
10.29.2021	567	54	60	1	60	50	47	26
11.15.2021	453	58	47	1	43	40	29	15
12.8.2021	352	80	41	1	34	40	30	24
12.22.2021	365	71	42	1	36	39	29	26
4.27.2022	260	169	52	2	31	43	39	36
8.12.2022	2	427	0	52	1	73	0	56
3.8.2023	432	81	68	2	78	60	69	61
5.19.2023	197	55	59	1	38	28	38	38
8.29.2023	177	218	60	5	33	55	39	57
9.12.2023	272	204	62	3	41	58	46	87
10.18.2023	191	183	47	2	31	43	29	34
12.14.2023	168	204	52	3	30	48	29	42
3.5.2024	219	60	144	26	30	80	41	72
4.29.2024	161	182	51	6	23	42	22	48
10.24.2024	185	195	44	3	30	44	26	52
11.7.2024	211	214	49	3	39	52	25	46
1.14.2025	132	176	51	9	22	46	26	48
4.9.2025	381	287	130	84	121	91	41	27
9.10.2025	377.0	185.0	55.0	0.0	66.0	73.0	48.0	57.0
